# Supplementary material for: A Critical View of the Application of the APEX Software (Aqueous Photochemistry of Environmentally-Occurring Xenobiotics) to Predict Photoreaction Kinetics in Surface Freshwaters
Source: Molecules. 2019 Dec 18;25(1):9. doi: 10.3390/molecules25010009 (PMC7017383; doi:10.3390/molecules25010009)
Supplement: Supplementary file 1 [file molecules-25-00009-s001.zip › 3.2.4_gcc-4.4.0.zip!/3.2.4_gcc-4.4.0/3.2.4_gcc-4.4.0/doc/octave/html/interpreter/Character-Arrays.html]

Character Arrays - Untitled


Next: Creating Strings,
Previous: Escape Sequences in string constants,
Up: Strings


---

### 5.2 Character Arrays

The string representation used by Octave is an array of characters, so
internally the string "dddddddddd" is actually a row vector of length 10
containing the value 100 in all places (100 is the ASCII code of "d"). This
lends itself to the obvious generalization to character matrices. Using a
matrix of characters, it is possible to represent a collection of same-length
strings in one variable. The convention used in Octave is that each row in a
character matrix is a separate string, but letting each column represent a
string is equally possible.

The easiest way to create a character matrix is to put several strings
together into a matrix.

```
     collection = [ "String #1"; "String #2" ];
```

This creates a 2-by-9 character matrix.

The function `ischar` can be used to test if an object is a character
matrix.

— Built-in Function: **ischar** (a)  
> Return 1 if a is a character array. Otherwise, return 0.

To test if an object is a string (i.e., a character vector and not a character
matrix) you can use the `ischar` function in combination with the
`isvector` function as in the following example:

```
     ischar(collection)
          => ans = 1
     
     ischar(collection) && isvector(collection)
          => ans = 0
     
     ischar("my string") && isvector("my string")
          => ans = 1
```

One relevant question is, what happens when a character matrix is
created from strings of different length. The answer is that Octave
puts blank characters at the end of strings shorter than the longest
string. It is possible to use a different character than the
blank character using the `string_fill_char` function.

— Built-in Function: val = **string\_fill\_char** ()  
— Built-in Function: old\_val = **string\_fill\_char** (new\_val)  
> Query or set the internal variable used to pad all rows of a character
> matrix to the same length. It must be a single character. The default
> value is `" "` (a single space). For example,
>
> ```
>           string_fill_char ("X");
>           [ "these"; "are"; "strings" ]
>                => "theseXX"
>                   "areXXXX"
>                   "strings"
> ```

This shows a problem with character matrices. It simply isn't possible to
represent strings of different lengths. The solution is to use a cell array of
strings, which is described in Cell Arrays of Strings.
